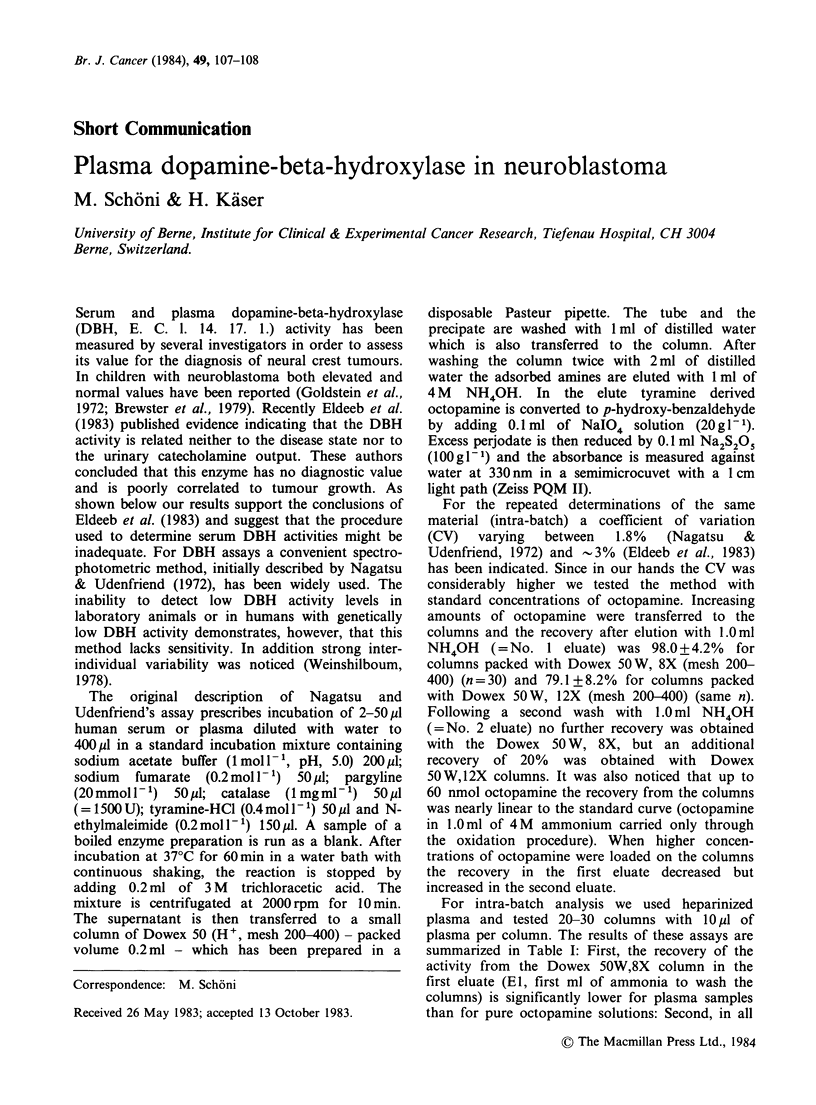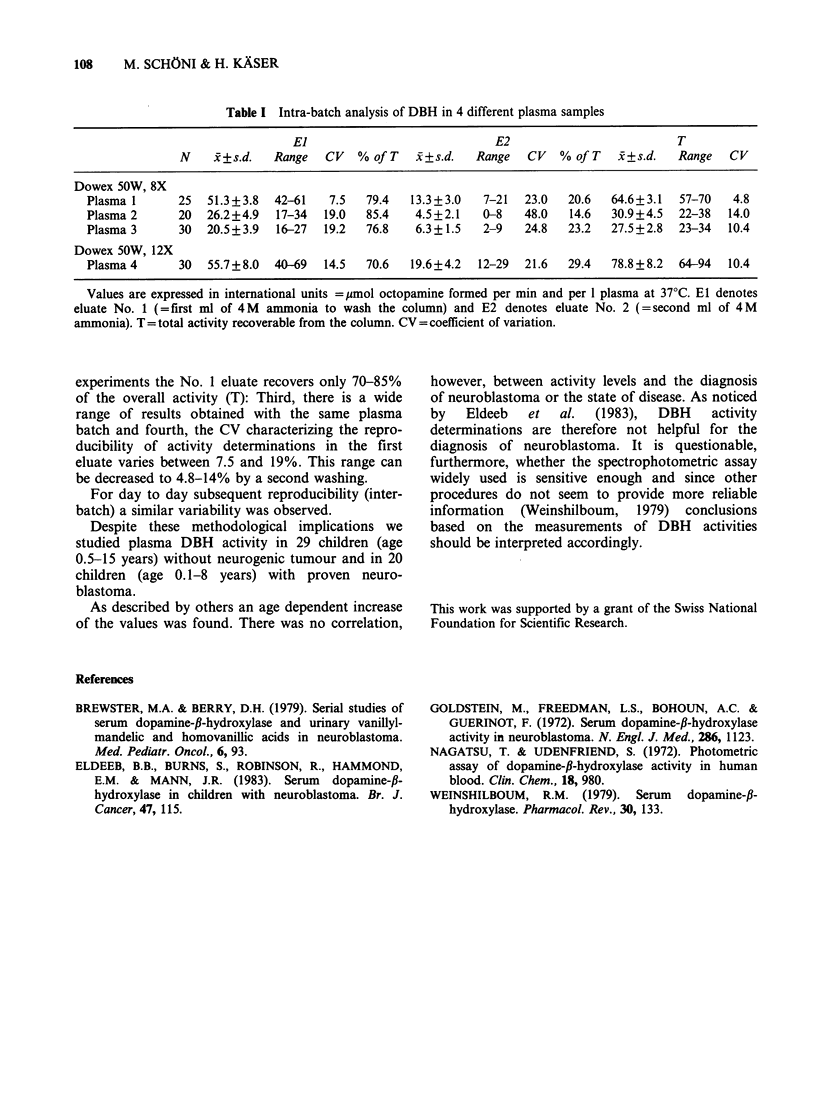# Plasma dopamine-beta-hydroxylase in neuroblastoma.

**DOI:** 10.1038/bjc.1984.17

**Published:** 1984-01

**Authors:** M. Schöni, H. Käser


Br. J. Cancer (1984), **49**, 107–108

### Short Communication

### Plasma dopamine-beta-hydroxylase in neuroblastoma

M. Schöni & H. Käser

*University of Berne, Institute for Clinical & Experimental Cancer Research, Tiefenau Hospital, CH 3004 Berne, Switzerland.*

Serum and plasma dopamine-beta-hydroxylase (DBH, E. C. 1. 14. 17. 1.) activity has been measured by several investigators in order to assess its value for the diagnosis of neural crest tumours. In children with neuroblastoma both elevated and normal values have been reported (Goldstein *et al.*, 1972; Brewster *et al.*, 1979). Recently Eldeeb *et al.* (1983) published evidence indicating that the DBH activity is related neither to the disease state nor to the urinary catecholamine output. These authors concluded that this enzyme has no diagnostic value and is poorly correlated to tumour growth. As shown below our results support the conclusions of Eldeeb *et al.* (1983) and suggest that the procedure used to determine serum DBH activities might be inadequate. For DBH assays a convenient spectrophotometric method, initially described by Nagatsu & Udenfriend (1972), has been widely used. The inability to detect low DBH activity levels in laboratory animals or in humans with genetically low DBH activity demonstrates, however, that this method lacks sensitivity. In addition strong inter-individual variability was noticed (Weinshilboum, 1978).

The original description of Nagatsu and Udenfriend's assay prescribes incubation of 2–50 $\mu l$ human serum or plasma diluted with water to 400 $\mu l$ in a standard incubation mixture containing sodium acetate buffer ($1 \, mol \, l^{-1}$, pH, 5.0) 200 $\mu l$; sodium fumarate ($0.2 \, mol \, l^{-1}$) 50 $\mu l$; pargyline ($20 \, mmol \, l^{-1}$) 50 $\mu l$; catalase ($1 \, mg \, ml^{-1}$) 50 $\mu l$ (= 1500 U); tyramine-HCl ($0.4 \, mol \, l^{-1}$) 50 $\mu l$ and N-ethylmaleimide ($0.2 \, mol \, l^{-1}$) 150 $\mu l$. A sample of a boiled enzyme preparation is run as a blank. After incubation at 37°C for 60 min in a water bath with continuous shaking, the reaction is stopped by adding 0.2 ml of 3 M trichloracetic acid. The mixture is centrifugated at 2000 rpm for 10 min. The supernatant is then transferred to a small column of Dowex 50 ($H^+$, mesh 200–400) – packed volume 0.2 ml – which has been prepared in a disposable Pasteur pipette. The tube and the precipate are washed with 1 ml of distilled water which is also transferred to the column. After washing the column twice with 2 ml of distilled water the adsorbed amines are eluted with 1 ml of 4 M $NH_4OH$. In the elute tyramine derived octopamine is converted to *p*-hydroxy-benzaldehyde by adding 0.1 ml of $NaIO_4$ solution ($20 \, g \, l^{-1}$). Excess perjodate is then reduced by 0.1 ml $Na_2S_2O_5$ ($100 \, g \, l^{-1}$) and the absorbance is measured against water at 330 nm in a semimicrocuvet with a 1 cm light path (Zeiss PQM II).

For the repeated determinations of the same material (intra-batch) a coefficient of variation (CV) varying between 1.8% (Nagatsu & Udenfriend, 1972) and ~3% (Eldeeb *et al.*, 1983) has been indicated. Since in our hands the CV was considerably higher we tested the method with standard concentrations of octopamine. Increasing amounts of octopamine were transferred to the columns and the recovery after elution with 1.0 ml $NH_4OH$ (= No. 1 eluate) was $98.0 \pm 4.2\%$ for columns packed with Dowex 50 W, 8X (mesh 200–400) ($n = 30$) and $79.1 \pm 8.2\%$ for columns packed with Dowex 50 W, 12X (mesh 200–400) (same *n*). Following a second wash with 1.0 ml $NH_4OH$ (= No. 2 eluate) no further recovery was obtained with the Dowex 50 W, 8X, but an additional recovery of 20% was obtained with Dowex 50 W,12X columns. It was also noticed that up to 60 nmol octopamine the recovery from the columns was nearly linear to the standard curve (octopamine in 1.0 ml of 4 M ammonium carried only through the oxidation procedure). When higher concentrations of octopamine were loaded on the columns the recovery in the first eluate decreased but increased in the second eluate.

For intra-batch analysis we used heparinized plasma and tested 20–30 columns with 10 $\mu l$ of plasma per column. The results of these assays are summarized in Table I: First, the recovery of the activity from the Dowex 50W,8X column in the first eluate (E1, first ml of ammonia to wash the columns) is significantly lower for plasma samples than for pure octopamine solutions: Second, in all

Correspondence: M. Schöni

Received 26 May 1983; accepted 13 October 1983.

© The Macmillan Press Ltd., 1984

**Table I** Intra-batch analysis of DBH in 4 different plasma samples

| | N | E1 $\bar{x}\pm s.d.$ | E1 Range | E1 CV | E1 % of T | E2 $\bar{x}\pm s.d.$ | E2 Range | E2 CV | E2 % of T | T $\bar{x}\pm s.d.$ | T Range | T CV |
|---|---|---|---|---|---|---|---|---|---|---|---|---|
| Dowex 50W, 8X | | | | | | | | | | | | |
| Plasma 1 | 25 | 51.3±3.8 | 42–61 | 7.5 | 79.4 | 13.3±3.0 | 7–21 | 23.0 | 20.6 | 64.6±3.1 | 57–70 | 4.8 |
| Plasma 2 | 20 | 26.2±4.9 | 17–34 | 19.0 | 85.4 | 4.5±2.1 | 0–8 | 48.0 | 14.6 | 30.9±4.5 | 22–38 | 14.0 |
| Plasma 3 | 30 | 20.5±3.9 | 16–27 | 19.2 | 76.8 | 6.3±1.5 | 2–9 | 24.8 | 23.2 | 27.5±2.8 | 23–34 | 10.4 |
| Dowex 50W, 12X | | | | | | | | | | | | |
| Plasma 4 | 30 | 55.7±8.0 | 40–69 | 14.5 | 70.6 | 19.6±4.2 | 12–29 | 21.6 | 29.4 | 78.8±8.2 | 64–94 | 10.4 |

Values are expressed in international units =μmol octopamine formed per min and per l plasma at 37°C. E1 denotes eluate No. 1 (=first ml of 4 M ammonia to wash the column) and E2 denotes eluate No. 2 (=second ml of 4 M ammonia). T=total activity recoverable from the column. CV=coefficient of variation.

experiments the No. 1 eluate recovers only 70–85% of the overall activity (T): Third, there is a wide range of results obtained with the same plasma batch and fourth, the CV characterizing the reproducibility of activity determinations in the first eluate varies between 7.5 and 19%. This range can be decreased to 4.8–14% by a second washing.

For day to day subsequent reproducibility (inter-batch) a similar variability was observed.

Despite these methodological implications we studied plasma DBH activity in 29 children (age 0.5–15 years) without neurogenic tumour and in 20 children (age 0.1–8 years) with proven neuroblastoma.

As described by others an age dependent increase of the values was found. There was no correlation, however, between activity levels and the diagnosis of neuroblastoma or the state of disease. As noticed by Eldeeb *et al.* (1983), DBH activity determinations are therefore not helpful for the diagnosis of neuroblastoma. It is questionable, furthermore, whether the spectrophotometric assay widely used is sensitive enough and since other procedures do not seem to provide more reliable information (Weinshilboum, 1979) conclusions based on the measurements of DBH activities should be interpreted accordingly.

This work was supported by a grant of the Swiss National Foundation for Scientific Research.

### References

BREWSTER, M.A. & BERRY, D.H. (1979). Serial studies of serum dopamine-β-hydroxylase and urinary vanillylmandelic and homovanillic acids in neuroblastoma. *Med. Pediatr. Oncol.*, **6**, 93.

ELDEEB, B.B., BURNS, S., ROBINSON, R., HAMMOND, E.M. & MANN, J.R. (1983). Serum dopamine-β-hydroxylase in children with neuroblastoma. *Br. J. Cancer*, **47**, 115.

GOLDSTEIN, M., FREEDMAN, L.S., BOHOUN, A.C. & GUERINOT, F. (1972). Serum dopamine-β-hydroxylase activity in neuroblastoma. *N. Engl. J. Med.*, **286**, 1123.

NAGATSU, T. & UDENFRIEND, S. (1972). Photometric assay of dopamine-β-hydroxylase activity in human blood. *Clin. Chem.*, **18**, 980.

WEINSHILBOUM, R.M. (1979). Serum dopamine-β-hydroxylase. *Pharmacol. Rev.*, **30**, 133.